# Cancer chemoprevention through dietary flavonoids: what’s limiting?

**DOI:** 10.1186/s40880-017-0217-4

**Published:** 2017-06-19

**Authors:** Haneen Amawi, Charles R. Ashby, Amit K. Tiwari

**Affiliations:** 1 Department of Pharmacology and Systems Therapeutics, College of Pharmacy and Pharmaceutical Sciences, University of Toledo, Toledo, OH 43560 USA; 2 Pharmaceutical Sciences, College of Pharmacy, St. John’s University, Queens, NY 11432 USA; 3 Department of Pharmacology and Experimental Therapeutics, College of Pharmacy and Pharmaceutical Sciences, University of Toledo, Toledo, OH 43614 USA

**Keywords:** Flavonoids, Chemoprevention, Silybin, Silymarin, Natural product drug development, Pharmacokinetic challenges

## Abstract

Flavonoids are polyphenols that are found in numerous edible plant species. Data obtained from preclinical and clinical studies suggest that specific flavonoids are chemo-preventive and cytotoxic against various cancers via a multitude of mechanisms. However, the clinical use of flavonoids is limited due to challenges associated with their effective use, including (1) the isolation and purification of flavonoids from their natural resources; (2) demonstration of the effects of flavonoids in reducing the risk of certain cancer, in tandem with the cost and time needed for epidemiological studies, and (3) numerous pharmacokinetic challenges (e.g., bioavailability, drug–drug interactions, and metabolic instability). Currently, numerous approaches are being used to surmount some of these challenges, thereby increasing the likelihood of flavonoids being used as chemo-preventive drugs in the clinic. In this review, we summarize the most important challenges and efforts that are being made to surmount these challenges.

## Background

**Fig. 1:**
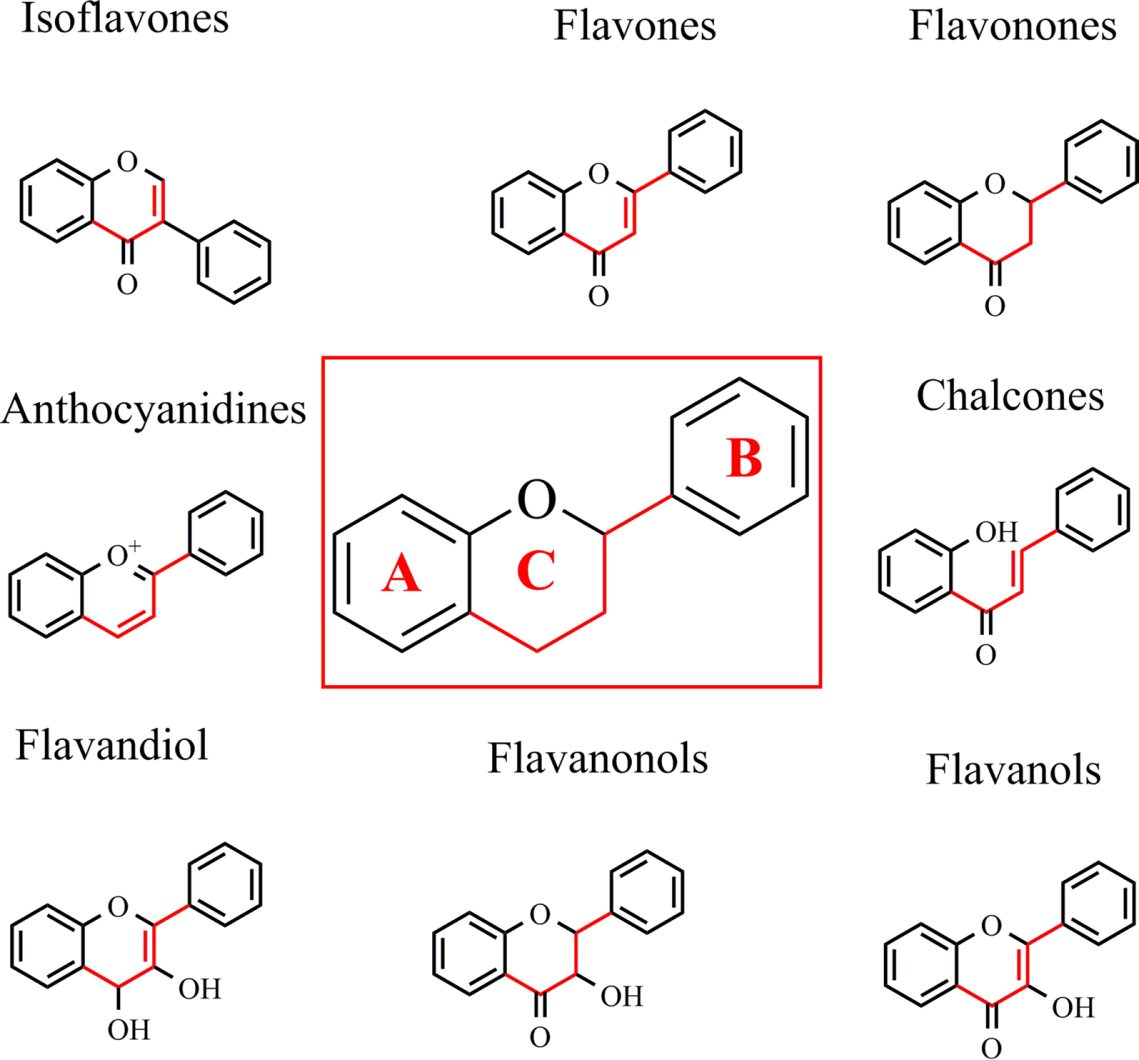
Subfamilies of flavonoids. Flavonoids include the following subfamilies: flavones, flavanols, isoflavones, flavonols, flavanones, and flavanonols, which differ in their ring substituents and extent of saturation

Dietary flavonoids are the most common polyphenols found in fruits, vegetables, flowers, chocolate, tea, wine, and other plant sources [1–3]. With more than 9000 members in this family, flavonoids can be divided into several subfamilies, including flavones, flavanols, isoflavones, flavonols, flavanones, and flavanonols that differ in their ring substituents and extent of saturation [4, 5]. However, all compounds in this family share the basic chemical structure consisting of two benzene rings connected by a 3-carbon bridge, forming a heterocycle (C6-C3-C6) [6] (Fig. 1). Flavonoids have been reported to have an excellent safety profile (no toxicity at up to 140 g/day), with no known significant adverse effects [7]. The pharmacological effects of flavonoids include antioxidant, anti-inflammatory, cardioprotective, hepatoprotective, antimicrobial, and anticancer [8, 9]. However, there are significant challenges associated with flavonoids related to their isolation, purification, and pharmacokinetic/pharmacodynamic (PK/PD) properties, which have limited their development into efficacious clinical drugs. Here, we discuss the challenges associated with the development of flavonoids for cancer chemoprevention and efforts to surmount these challenges.

## Flavonoids in cancer chemoprevention

Increasing evidence from both epidemiological and laboratory studies suggests that the dietary intake of flavonoids reduces the risk of developing certain types of cancers [10]. Several types of flavonoids have been identified as having antiproliferative efficacy in various cancers, including silymarin, genistein, quercetin, daidzein, luteolin, kaempferol, apigenin, and epigallocatechin 3-gallate [11, 12]. These aforementioned compounds have been reported to have anticancer and preventive effects against prostate [13], colorectal [14], breast [15], thyroid [16], lung [17], and ovarian [18] cancers, among others [19–21]. Their chemopreventive efficacy is mediated by (1) inhibiting the development of new cancer cells; (2) preventing carcinogens from reaching their activation sites; and (3) decreasing the toxicity of certain compounds by inhibiting their metabolism [22–24]. The molecular mechanisms by which flavonoids produce their anticancer and preventive effects include (1) induction of apoptosis [14, 25, 26]; (2) cell cycle arrest at G_1_ or G_2_/M phase by inhibiting key cell cycle regulators such as cyclin-dependent kinases (CDKs) [27, 28]; (3) inhibition of metabolizing enzymes (notably cytochromes P450 [CYPs]), which inhibits the activation of numerous carcinogenic compounds [29]; (4) inhibition of reactive oxygen species formation primarily by activation of phase II metabolizing enzymes [26, 30, 31]; and (5) inhibition of vascular endothelial growth factor (VEGF)- and basic fibroblast growth factor (bFGF)-mediated angiogenesis [32–34]. In addition, some flavonoids have been shown to significantly inhibit multidrug resistance, which is responsible for cancer relapse and chemotherapy failure [35, 36]. However, some flavonoids have specific mechanisms of action that are not characteristic of the flavonoid family. For example, the isoflavones genistein and diadzein have been shown to significantly inhibit cancer growth and proliferation [37–39]. Due to their structural similarity with estrogen, genistein and diadzein have been reported to have significant preventive efficacy against breast cancer [40, 41]. Another interesting flavonoid is silybin, which has antioxidant and hepatoprotective efficacy [42–44]. However, in vitro and in vivo preclinical studies in the last decade indicate that silybin also has antiproliferative efficacy and, as a result, subsequent phase I and II clinical studies have been conducted [45–47]. Silybin has a number of pharmacological properties that may explain its anticancer efficacy, such as inhibition of (1) tumor necrosis factor (TNF)-induced activation of nuclear factor kappa B (NF-κB) where it inhibits the phosphorylation and proteolytic degradation of nuclear factor of kappa light polypeptide gene enhancer in B cell inhibitor, alpha (IκBα) to NF-κB (active form) [48]; (2) tyrosine kinases [49]; (3) androgen receptors [50]; and (4) the epithelial-to-mesenchymal transition embryonic pathways [51–54]. Another flavonoid, quercetin, is a potent antioxidant that is present in natural sources such as berries, onions, apples, and red wine [3, 55]. Quercetin’s anticancer efficacy in colon cancer and neurogliomas results from activating the novel cell death pathway, autophagy (type II programmed cell death), and mitogen-activated protein kinases (MAPK or extracellular signal-related kinase [ERK]) signaling pathways [56–58]. Accordingly, several studies on flavonoids support the potential role of flavonoids in both cancer treatment and prevention [1]. Currently, a variety of flavonoid formulations are present in dietary supplements such as milk thistle and red clover extracts [59]. However, none of the above mentioned flavonoids have been approved for clinical use.

## Challenges in flavonoids in cancer chemoprevention development

**Fig. 2:**
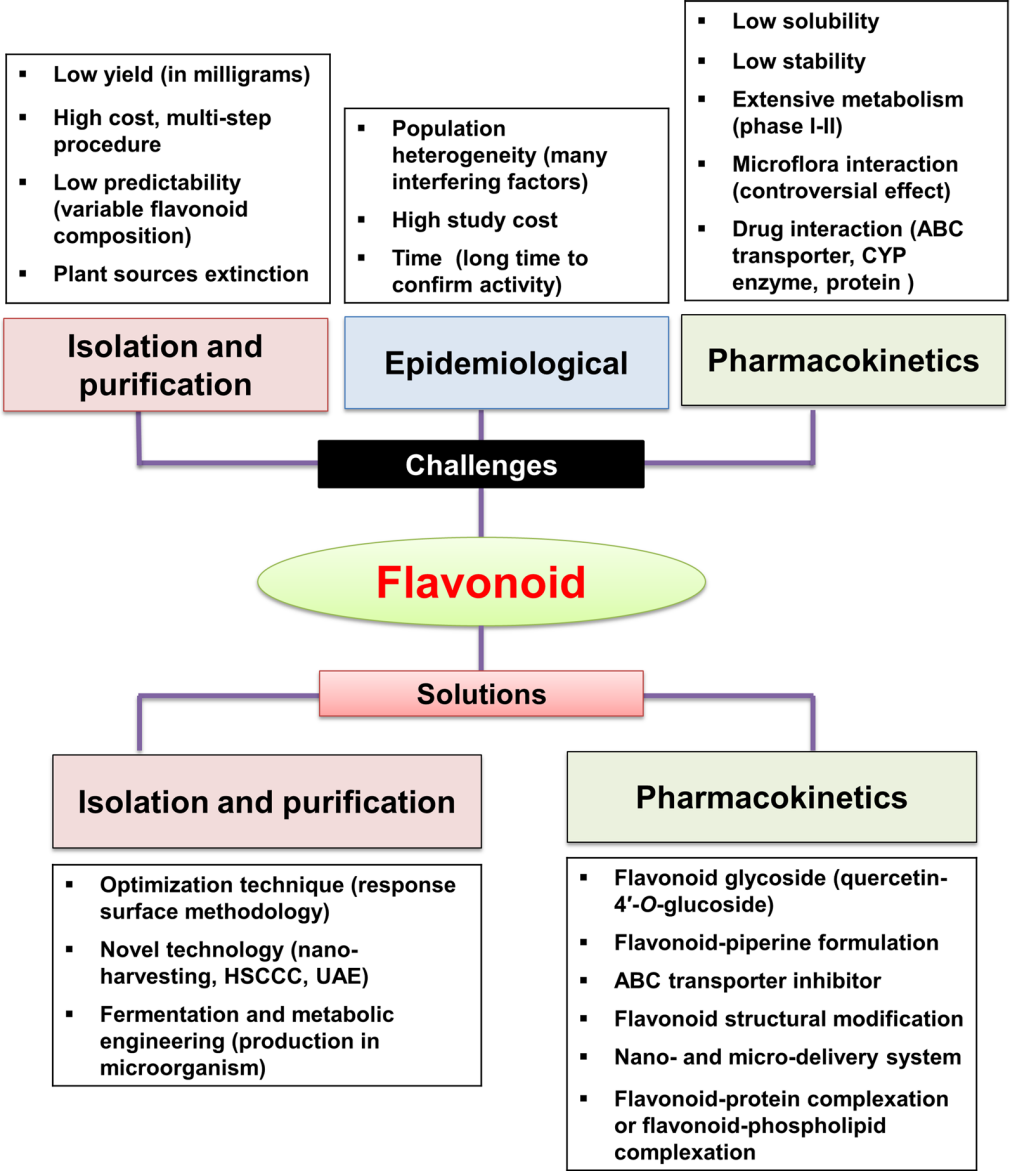
Challenges associated with flavonoid development and possible approaches to overcome their use as chemopreventive agents. *ABC*: ATP-binding cassette transporters, *CYP*: cytochrome P450. *HSCCC*: high-speed counter-current chromatography, *UAE*: ultrasound-assisted extraction

Despite preclinical evidence suggesting that flavonoids have anticancer and preventive efficacy, there are numerous problems that have impeded the development of dietary flavonoids as approved drugs for clinical use. There are challenges associated with demonstrating the effect of flavonoids in reducing the risk of certain cancer, e.g., the cost and time needed for epidemiological studies, the isolation and purification of flavonoids from their natural sources, and PK issues, among others. These challenges are discussed below, and a summary of these issues is also presented in Fig. 2.

### Isolation and purification challenges

One of the major challenges in the extraction of flavonoids from their original plant sources resides in the fact that these compounds are present at very low levels (from micrograms to milligrams per kg of plant masses). Indeed, the continuous extraction of these compounds could result in the extinction of the plant source (assuming extraction is faster than replenishing new plants), disrupting whole plant communities [60, 61].

As with other plant products, flavonoids are usually present in plants as a complex with other compounds that produce their effects in concert. In addition, other secondary metabolites, minerals, vitamins, and fibers are also complexed with flavonoids from the same source [59]. Therefore, the sum of the constituents in the plant may be responsible for the observed anticancer efficacy, as opposed to more than one flavonoid alone [62]. This complexation of flavonoids makes it difficult to isolate and identify the exact molecule that is producing specific pharmacological effects. Also, following the identification of the active flavonoid, its subsequent isolation and purification from other compounds, using analytical methods, is a multistage procedure. A combination of several technologies can be used for isolation of specific compounds, including solvent extraction, column chromatography, medium-pressure liquid chromatography, vacuum column chromatography, and preparative high performance liquid chromatography (HPLC) [63, 64]. The application of such procedures is a time-consuming process that can be associated with high costs [65]. Additionally, even with the application of such complex techniques, the yield of extracted compounds is typically very low as several kilograms of the plant produce less than 1 g of the isolated compounds in some cases [66]. An important factor that limits the extraction yield is the complex nature of biosynthetic pathways for flavonoids in plants. These pathways are considered to be one of the most complex biosynthetic pathways and result in variable flavonoid composition at different growth stages of the plant and under different environmental conditions [67, 68]. The variation in flavonoid composition decreases the predictability of flavonoid yields during extraction and results in inconsistent data after each extraction [69]. Another limitation in the extraction of flavonoids is that these compounds are usually labile, subjecting them to a high level of degradation or alteration in their chemical structures and subsequent loss of activity during purification [70]. Therefore, harvesting flavonoids from their plant natural sources, using the current applied methodologies, can be time-consuming, highly expensive, associated with very low yields and wasteful.

### Epidemiological challenges

The potential therapeutic effects (as chemopreventive compounds) of natural products such as flavonoids can be ascertained to some extent from epidemiological studies, including retrospective meta-analysis [71], prospective observational studies [72], and/or prospective interventional studies [73, 74]. The data from epidemiological studies depend on the population of individuals that have ingested the specific compound. Thus, for this review, the population of interest would consist of those individuals that have taken dietary flavonoids to prevent cancer. It is time-consuming to collect the data and have it categorized, analyzed, and associated with the presence or absence of flavonoids. The data is often skewed due to lack of adherence data in the population using specific flavonoids. Also, the length of exposure is very long, where some changes in the amount and type of exposure can occur rapidly in the population, making the conclusions of the study invalid. Another important limitation is that the population is usually exposed to many heterogeneous factors that can significantly affect health outcomes, including the development of cancer. Such factors can result in conflicting data and decrease the certainty of conclusions about the effect of flavonoids in cancer chemoprevention. For example, the data from epidemiological studies that were used to determine the correlation between the intake of dietary flavonoids and the risk of developing colorectal cancer (CRC) were controversial and inconsistent [75, 76]. Some studies suggested that flavonoid consumption was significantly correlated with a low CRC risk [77–79], whereas other studies did not report a significant correlation between the intake of flavonoids and CRC risk [80–82]. Therefore, the type of dietary flavonoid that is ingested, the size and heterogeneity of population, and the design of the study can affect the interpretation of results from studies assessing the effectiveness of flavonoids in preventing cancer. The validation of the conclusions derived from appropriately designed epidemiological studies requires conducting expensive studies using large populations, which further limits the development of flavonoids as drugs.

### PK challenges

Flavonoids typically have an unsuitable PK profile [83, 84] (i.e., absorption, distribution, metabolism, excretion, and toxicity [ADMET]), characterized by low solubility, poor oral absorption, and extensive hepatic metabolism by phase I and II enzymes [85–87]. Flavonoids are usually ingested with other foods components, resulting in the complexation or precipitation of flavonoid compounds, thus limiting their absorption and bioavailability [88, 89]. Furthermore, flavonoids can undergo significant metabolism via de-glycosylation prior to their absorption in the small intestine epithelial cells [90, 91]. In vivo, flavonoids are substrates for glucuronidation, sulfation, and O-methylation [92], resulting in inert, polar complexes that are rapidly excreted in urine [85]. Furthermore, the unabsorbed form can reach the colon and undergo degradation by the intestinal microflora by ring fission [93–95], reduction [96], or hydrolysis [97]. For example, only 20%–30% of an oral dose of quercetin is bioavailable [98]. The incubation of quercetin under normal physiological conditions (Hanks’ Balanced Salt solution, pH 7.4) results in its degradation within 6 h [99]. The anticancer effect of the flavonoid silybin is limited by its extensive metabolism and low oral absorption [100, 101]. Flavan-3-ols have been shown to be completely degraded after 8 h of exposure to simulated intestinal secretions [102]. These aforementioned PK liabilities represent significant barriers for the clinical development of flavonoids, as the required in vivo levels cannot be achieved even with high oral doses [103, 104]. In addition, the ingestion of higher doses of flavonoids for more effective antiproliferative effect may produce proliferative and inflammatory responses [105, 106]. Finally, flavonoids are known to affect the bioavailability and efficacy of many drugs due to their multiple in vivo interactions. For example, certain flavonoids can affect CYPs [107] and conjugation enzymes [108], other enzymes (α-amylase [109] and α-glucosidases [110]), bovine hemoglobin [111], multidrug resistance transporters [112], colonic microflora [113], and plasma proteins [114, 115].

#### ATP-binding cassette drug transporter interactions

The ATP-binding cassette (ABC) transporter superfamily consists of important members that mediate not only PK alterations (i.e., ADMET), but also multidrug resistance (MDR) to numerous antineoplastic drugs, including flavonoids, that are substrates for these transporters, resulting in chemotherapeutic failure [116–119]. Important members include P-glycoprotein (P-gp or multidrug resistance protein family 1 [MDR1]), breast cancer-resistant protein (BCRP or ATP-binding cassette sub-family G member 2 [ABCG2]), and multidrug resistance protein family C member 1 (ABCC1 or multidrug resistance-associated protein 1 [MRP1]) [120]. The transporters are located on the cell membrane with two transmembrane domains that can recognize different compounds and form channels within the membrane to efflux these compounds [121]. The efflux of the compounds requires the hydrolysis of ATP which provides the energy required for efflux of substrates [122]. ABC transporters have a ubiquitous distribution throughout the body, although they are present in high densities in tissues that have a barrier function, such as the gastrointestinal tract, reproductive organs, kidney, liver, and blood–brain barrier [123]. It is well established that ABC transporters play a critical role in regulating drug absorption, distribution, and excretion, which can decrease their bioavailability and thus their efficacy [124, 125].

Several reports investigated the possible interaction of flavonoids with ABC transporters. Flavonoids, such as flavones (e.g., apigenin and chrysin), isoflavones (e.g., biochanin A and genistein), flavonols (kaempferol), and flavanones (naringenin) have been reported to inhibit the efflux function of ABC transporters, such as ATP-binding cassette subfamily B member 1 (ABCB1) and ABCG2 [112, 126, 127]. The inhibition of ABC transporters by certain flavonoids can have advantages and disadvantages. The inhibition of ABC transporters can increase the bioavailability of some poorly available drugs, thereby potentially augmenting the absorption, distribution, bioavailability, and efficacy of certain drugs, including antineoplastics. Such inhibition can be used to overcome multidrug resistance and chemotherapy failure [126]. For example, the isoflavinoids medicarpin and millepurpan significantly induce apoptosis in multidrug-resistant P388 leukemia cells and overcome the resistance mechanisms [128]. Epigallocatechin-3-gallate, at a dose of 10 mg/kg bodyweight by intragastric gavage as a suspension in 0.2% agar, once a day for 10 days, significantly decreased the expression of P-gp, which increased the plasma levels of atorvastatin and verapamil in male Wistar rats, potentiating their pharmacological actions [129].

However, the inhibition of ABC transporters by specific flavonoids can potentiate the toxicity of certain ABC substrates and elicit unexpected adverse or toxic effects of these substrates such as antimicrobials [130, 131], immunosuppressants [132], cardiovascular [133–135], and chemotherapeutic drugs [136, 137]. A recent report indicated that some flavonoids (e.g., genistein and glyceollin) also interact with other ABC transporters such as ABCC2 (MRP2) [138]. In addition, certain flavonoids are substrates for ABC transporters, thereby limiting their absorption from the gastrointestinal tract, distribution to body tissues and organs, and, ultimately, their bioavailability [139]. Polymorphisms in the ABC transporter genes can directly affect the PK profile of flavonoids. For example, a recent study showed that the ABCB1 C3435T polymorphism significantly altered the bioavailability and plasma levels of silybin. Patients with CC or CT polymorphisms in the *ABCB1* gene have twice the plasma levels of silybin compared to patients with the TT polymorphism [140]. Detailed interactions of flavonoids with CYPs are reviewed elsewhere [108, 137].

#### CYP interactions

CYPs play a significant role in the biotransformation of xenobiotic and endogenous compounds [141]. It is well established that CYPs play a crucial role in phase I metabolism, typically bio-transforming molecules to more polar entities and increasing the likelihood they will be substrates for phase II metabolism. Flavonoids have been reported to significantly inhibit the activities of CYPs [109]. This inhibition is mediated by either a reduction in the level of CYPs or direct binding of flavonoids to their active sites [110]. CYP 3A4 is one of the most important CYP isoforms and is involved in the metabolism of many clinically used drugs [142]. Several types of flavonoids, such as quercetin, kaempferol, naringenin, and apigenin have been shown to have inhibitory effects on the activities of CYPs, primarily CYP 3A4 (both in vivo and in vitro) [143, 144]. This inhibition increases the half-lives and the plasma concentrations of many drugs that are substrates for CYPs, which can potentiate their adverse effects and/or toxicity. For example, the adverse effects of certain calcium channel blockers, statins, antihistamines, protease inhibitors, and immunosuppressants can be significantly potentiated by specific flavonoids [145]. In addition to the inhibition of CYP 3A4, flavonoids were reported to inhibit other CYP isoforms, such as CYP subfamily 1 isoforms (CYP 1A1, CYP 1A2, and CYP 1B1), which are significantly involved in carcinogenesis [146]. The two isoflavones, formononetin and biochanin A, significantly inhibit CYP 1A2 in both human and rat liver microsomes in vitro. Formononetin also significantly inhibits CYP 2D6, and biochanin A also inhibits human CYP 2C9 [147]. CYP 1B1 is inhibited by flavone [148], chrysin [148], apigenin [148], genistein [148], luteolin [149], quercetein [149], galangin [149], myricetin [150], and many others. CYP 1A1 is irreversibly inhibited by the binding of two flavones (3-flavone propargyl etherE and 7-Hydroxy flavone) [151]. Finally, *CYP* gene expression was inhibited by the flavonoids, apigenin [152], tangeretin [153], diadzein [154], silybin [155], and others. Detailed interactions of flavonoids with CYPs are reviewed elsewhere [156].

#### Intestinal microflora interactions

Following the oral administration of flavonoids, it is possible that a significant percentage can reach the colon and be subjected to degradation by microflora, as well as enterohepatic circulation, depending on the compound [157]. The colonic microflora is the most abundant and diverse part of the microbiome in humans [158]. These microorganisms have been shown to biotransform certain drugs to metabolites, thereby altering their efficacies and toxicities [159–161]. They also act as a protection barrier involved in the defense against pathogens and toxic xenobiotics. The colonic microflora also reduces cholesterol absorption and increases mucus secretion in the gut [162, 163]. The role of the colonic microflora on the absorption, metabolism, and bioavailability of flavonoids remains to be delineated [164]. It has been reported that unabsorbed flavonoids can be biotransformed to small phenolic compounds that have similar effects, but improved bioavailability, compared to the parent compound [165]. In contrast, the colonic microflora can extensively metabolize (via cleaving the heterocycle break) flavonoids via the enzymes glucuronidase and sulphatase, producing metabolites that are primarily inert polar compounds that are rapidly excreted [164, 166–168]. Some flavonoids (e.g., apigenin, genistein, naringenin, and kaempferol) are more likely to undergo microflora degradation compared with others, resulting in lower bioavailability [169]. Recent reports indicated that certain flavonoids can inhibit intestinal microflora and their associated fermentation processes [170]. Both bacterial β-glucosidase and α,β-galactosidase were inhibited by ellagitannins and flavan-3-ols from raspberry extracts [171].

Furthermore, the use of antibiotics should be monitored when using along with flavonoids as they can alter the composition of the gut microflora, which ultimately affects the bioavailability of specific flavonoids [172]. Thus, the huge diversity in the structures of flavonoids, as well as the microbial composition of gastrointestinal tract, can lower the predictability of the types of interactions that occur, as well as the effect of the resultant compounds and their permeability.

#### Other PK challenges

The poor chemical stability of flavonoids has been shown to adversely affect PK and limit their utility. Several factors, such as oxygen exposure, temperature, light, ultraviolet radiation, and pH, were shown to reduce flavonoid stability and result in its subsequent degradation [173]. Indeed, increased oxidation due to the presence of oxygen significantly alters cranberry flavonoid stability [174]. Temperature is another factor that needs to be optimized upon the extraction, purification, and storage of flavonoids. For example, the highest yield of phenolic flavonoids from the pericarp of litchi fruit extraction was achieved at 45 °C–60 °C, whereas other temperatures resulted in significantly lower yields and substantial degradation of flavonoids [175]. Light exposure can also alter flavonoid biosynthesis and its biological activities. The optimum antioxidant activity of total flavonoids in the plant *Halia bara* was at a light wavelength of 310 μmol/m^2^ s^1^. Other tested wavelengths reduced the biosynthesis and antioxidant activity of the total flavonoids [176]. Additionally, different pH values can result in distinct yields and activities of flavonoids. A pH range of 3–4 produced the highest yield and bioactivity in phenolics from litchi fruit pericarp [175]. The chemical structure itself and the type of substitution on the flavonoid rings can also alter the chemical stability. For example, the degradation of flavonols when exposed to long wavelength ultraviolet A radiation was increased with more ring substitutions [177].

The interaction of dietary flavonoids with fiber is another issue, which may significantly affect the absorption and bioavailability of flavonoids [178, 179]. Fibers can delay the absorption of flavonoids from the intestine by two major mechanisms. First, dietary fiber forms complexes with flavonoids, trapping flavonoids in their matrix; second, the fiber can significantly enhance gastric fluid viscosity, which restricts the gastric mixing process, thereby further decreasing the absorption of flavonoids [179, 180].

## Approaches to surmount flavonoid PK/PD and other barriers

There are a number of approaches that are being investigated to improve and surmount the challenges associated with clinical use of dietary flavonoids (Fig. 2).

### Improving purification and isolation yields

As mentioned earlier, the current traditional isolation and purification techniques usually result in low extraction yield of flavonoids that does not justify the high extraction cost. However, optimization of the conditions in these traditional extraction methods may increase the extraction yield of flavonoids. Response surface methodology (RSM) was applied to optimize flavonoid extraction using ethanol from herbal medicines like *Citrus aurantium* L. var. amara Engl [181] and Chinese Huangqi [182]. RSM is a mathematical and statistical method for designing experiments [183]. RSM significantly increased the yield of flavonoids from Chinese Huangqi when the extraction parameters were optimized as follows: ethanol concentration, 52.98%; extraction time, 2.12 h; extraction temperature, 62.46 °C; and a liquid–solid ratio of 35.23 [182]. However, such optimization is required for each plant source of flavonoids and can be time-consuming. Therefore, several novel technologies can be applied to reduce the cost and the loss of extracted flavonoids from their natural sources. One of these is the use of high-speed, counter-current chromatography that has been reported to be of lower cost and produce higher yields compared with other technologies [63]. Another technology that has recently emerged is nano-harvesting, where nanoparticles are used to harvest flavonoids from their sources [184]. The nanoparticles enter the plant structures and are released to bind to the targeted compounds and carry them outside the cells without harming the plants. This technique eliminates the use of organic solvents, allows for continuous production of flavonoids, and has opened a new era in natural product extraction methodologies [185]. The ultrasound-assisted extraction method has been purported to increase extraction efficiency and reduce the required time for extraction [181, 186, 187].

As mentioned in the challenges section, the extraction of certain compounds from the plant source can significantly harm plant communities. Therefore, the microbial production of plant natural products, such as flavonoids, at an industrial scale, is currently an attractive alternative approach [61, 188]. This approach has the potential to preserve the environmental resources and use economical stocks associated with less energy use and waste emission. Currently used microorganisms include *Escherichia coli* [189, 190] and *Saccharomyces cerevisia*e [191, 192]. The engineering and synthetic biology of microorganisms encourage the return to natural compounds as promising anticancer agents [61, 193].

### Overcoming PK challenges

There are a number of approaches or strategies that can be used to surmount factors that lower the bioavailability of flavonoids. For example, the formulation of flavonoids as certain types of glycosides can result in enhanced bioavailability compared with the flavonoid alone or other types of glycosides [194]. These glycosidic derivatives are substrates for certain intestinal epithelial transporters, which would increase their absorption [195]. The administration of quercetin-4′-*O*-glucoside resulted in a plasma level that was 5 times higher than of quercetin-3-*O*-rutinoside. Therefore, the conversion of quercetin glycosides into glucosides can be considered an approach to improve flavonoid bioavailability [194]. Another strategy involves adding piperine to the flavonoid formulations. The use of bioenhancers, such as piperine, which is an amide alkaloid from the plants of the *Piperaceae* family, is another approach [196]. Piperine significantly inhibits the conjugation of various flavonoid compounds such as quecetin [197] and epigallocatechin-3-gallate [198] by certain UDP-glucuronosyltransferase phase II enzymes, decreasing their metabolism and increasing bioavailability [197–199]. The use of more specific novel ABC transporter blockers such as lapatinib, nilotinib, or specific small interfering RNA is another option, provided that they do not produce intolerable adverse effects, for flavonoids whose bioavailability is limited by certain ABC transporters [200]. In addition, the efficiency of modulators of the intestinal microflora can be considered to improve the flavonoid bioavailability. Such modulation could be achieved by the use of antibiotics or other formulation products that can bypass the gut microbiome [201].

One of the most important strategies to optimize the PK/PD parameters is the modification of the flavonoid structure to produce novel derivatives. These compounds would contain the basic pharmacophore of the parent compound to retain their desired effects. Methyl- and hyro-sliybin derivatives have been reported to be 10-fold more potent than the parent compound, sylibin [202–205]. The introduction of hydrophobic functional groups (e.g., ethyl substitution) on the hydroxyl (OH) groups in quercetin significantly enhances its stability by preventing oxidative degradation of the hydroxyl groups [206]. Furthermore, the hydrophobic substitutions increase lipophilicity (quercetin’s clogP = 2, hydrophobic derivatives clogP = 3–12), which increases penetrability through biological membranes (bioavailability was increased from 10.7% for quercetin to 18.8% for one of its derivatives) [206]. It has also been shown that blocking some groups (e.g., C3 hydroxyl and C7 hydroxyl groups) in quercetin by the introduction of the lipophilic moiety pivaloxymethyl (POM) enhances its solubility, decreases its metabolism, enhances stability (half-life increased from 10 h for quercetin to >72 h for its quercetin-POM conjugates at pH 7.4), and increases its effectiveness by preventing chemical and metabolic hydrolysis [207]. An epoxypropoxy flavonoid derivative (MHY336), by inhibiting the enzyme topoisomerase II enzyme, exhibited significant potency against the prostate cancer cell lines LNCaP, PC-3, and DU145 [208].

An area that has shown significant growth is the development and use of micro- and nanodelivery systems to maximize the bioavailability of flavonoids [209–212]. One of these approaches involves the use of kinetically stable nanoemulsion technology, where the lipophilic flavonoids can be prepared as emulsions consisting of extremely small particle size (<200 nm). The emulsified flavonoids are released slowly over time, allowing for a higher surface area for absorption, ultimately improving their absorption and bioavailability after oral administration [213]. Another approach is the advanced delivery system with nano-crystal, self-stabilized pickering emulsions that has been reported to increase the delivery of some flavonoids including silybin [214]. Formulating flavonoids as a povidone-mixed, micelle-based microparticle has been shown to significantly enhance their release and PK profile [215]. The encapsulation of the flavonoid quercetin in Zein nanoparticles increases effectiveness in a mouse model of endotoxemia [216].

Flavonoid complexing with protein has been shown to increase flavonoid stability in vitro [217, 218]. Several studies suggest that this characteristic of flavonoids can be used to enhance their chemical stability [219–221]. The overall stability of the grape skin-derived anthocyanine extracts was enhanced when complexed with the proteins α- and β-casein [221]. Furthermore, studies indicate that other milk-derived proteins (e.g., whey proteins and β-lactoglobulin), when used as carriers, also enhance the chemical stability of anthocyanin extracts and allow for their incorporation as food formulations [219]. The complexation of flavonoids with phospholipids has been reported to enhance their bioavailability [222]. The amphiphilic nature of phospholipids helps in enhancing the passage of compounds across the membranes [223]. Indeed, the complexing of the flavonoid quercetin with phospholipid (phosphatidylcholine) to form a quercetin-phospholipid complex significantly improved the PK parameters (maximum serum concentration that a drug achieves and area under the curve) of quercetin in rats compared with quercetin alone [224].

## Conclusions

The preclinical anticancer effect of certain flavonoids suggests that the flavonoids may prevent certain types of cancer. However, the development of flavonoids is limited by their poor extraction yield, complicated extraction methods, the cost and difficulties of epidemiological studies, and their unfavorable PK characteristics. Versatile strategies are being applied to overcome such limitations. Future studies are required to determine whether these strategies can be applied economically and safely. The modulation of phase II metabolism and intestinal microflora can affect the metabolism, bioavailability, and toxicity of other drugs. It also can modulate the availability of dietary minerals and vitamins, thereby having potential impacts on health. Consequently, it may be more preferable to conduct research directed towards new delivery systems, such as nano-emulsions and nanoparticles. These delivery systems should be expected to have enhanced target specificity and safety. However, the cost of developing natural products and applying these strategies should be considered in the light of the cost of currently available synthetic compounds.
