# Erratum to: Cancer chemoprevention through dietary flavonoids: what’s limiting?

**DOI:** 10.1186/s40880-017-0237-0

**Published:** 2017-08-30

**Authors:** Haneen Amawi, Charles R. Ashby, Amit K. Tiwari

**Affiliations:** 1 Department of Pharmacology and Systems Therapeutics, College of Pharmacy and Pharmaceutical Sciences, University of Toledo, Toledo, OH 43560 USA; 2 Pharmaceutical Sciences, College of Pharmacy, St. John’s University, Queens, NY 11432 USA; 3 Department of Pharmacology and Experimental Therapeutics, College of Pharmacy and Pharmaceutical Sciences, University of Toledo, Toledo, OH 43614 USA

## Erratum to: Chin J Cancer (2017) 36:50 DOI 10.1186/s40880-017-0217

After publication of the original article [1], it is noticed that the cited author list and Google Scholar link in Reference 184 are not correct. Revised Reference 184 is as follows:

“184. Khan MA, Rankin SE, Littleton JM and Knutson BL. 471914 nanoharvesting of polyphenolic flavonoids from solidago nemoralis hairy root cultures using functionalized mesoporous silica nanoparticles. 2016 AIChE annual meeting 2016. San Francisco: American Institute of Chemical Engineers; 2016.

https://scholar.google.com/citations?view_op=view_citation&hl=en&user=F4lbzUkAAAAJ&citation_for_view=F4lbzUkAAAAJ:geHnlv5EZngC”.

## References

[1] Amawi H, Ashby CR, Tiwari AK (2017). Cancer chemoprevention through dietary flavonoids: what’s limiting?. Chin J Cancer.

